# Trends in the global burden of cystic echinococcosis among children and adolescents from 1990 to 2021: An analysis based on the Global Burden of Disease Study 2021

**DOI:** 10.1371/journal.pntd.0013658

**Published:** 2025-10-30

**Authors:** Tong Liu, Guangfu Li, Hangshuai Qu, Runle Li, Xuequan Wang, Feng Tang

**Affiliations:** 1 Research Center for High Altitude Medicine, Key Laboratory of the Ministry of High Altitude Medicine, Qinghai university, Xining, Qinghai, China; 2 Key Laboratory of Applied Fundamentals of High Altitude Medicine, (Qinghai-Utah Joint Key Laboratory of Plateau Medicine), Qinghai university, Xining, Qinghai, China; 3 Laboratory for High Altitude Medicine of Qinghai province, Qinghai university, Xining, Qinghai, China; 4 Department of Pharmacy, Taizhou hospital of Zhejiang Province Affiliated to Wenzhou Medical University, Taizhou, Zhejiang, China; 5 Department of Public laboratory, Taizhou Hospital of Zhejiang Province Affiliated to Wenzhou Medical University, Taizhou, Zhejiang China; 6 Department of Radiation Oncology, Taizhou Hospital of Zhejiang Province Affiliated to Wenzhou Medical University, Taizhou, Zhejiang, China; National Institute of Allergy and Infectious Diseases Division of Intramural Research, UNITED STATES OF AMERICA

## Abstract

**Background:**

Cystic echinococcosis (CE), caused by ***Echinococcus granulosus***, is a zoonotic disease with major global social and economic impacts. Research on its burden in children and adolescents remains limited. This study evaluates the global CE burden from 1990 to 2021 and projects future trends, supporting WHO NTD Roadmap goals aimed at enhancing control in 17 high-endemic countries by 2030.

**Methods:**

Using the Global Burden of Disease (GBD) database, we assessed prevalence, incidence, deaths, DALYs, YLDs, and YLLs due to CE in individuals aged 0–19 at global, regional, and national levels. We computed age-standardized rates (ASRs) and estimated annual percentage changes (EAPCs). Additional analyses included joinpoint regression, inequality measures, frontier and decomposition analysis, age-period-cohort (APC) modeling, Socio-demographic Index (SDI) correlations, and future trend prediction.

**Results:**

Over 32 years, the global CE burden declined overall, though disparities persisted. Low SDI regions had high ASPR, ASIR, and ASMR. In 2021, global ASIR was 1.12 per 100,000, ASPR was 3.71, and ASMR was 0.01. Moldova had the highest ASPR; Iceland the lowest. East Asia saw growth in ASPR and ASIR. South Sudan had the highest ASMR; Ethiopia had the highest ASDR. Females showed higher ASPR and ASIR; males had higher ASMR. A strong negative correlation was observed between SDI and health indicators. Population changes primarily influenced ASPR. Frontier analysis indicated elevated ASMR/ASDR in some low-SDI nations and rising trends in certain high SDI countries. Age-specific prevalence increased with age. Projections suggest a slow decline in CE burden over the next 25 years, though some countries will remain severely affected.

**Conclusions:**

The global CE burden in children and adolescents decreased from 1990 to 2021, yet challenges remain, especially in low-SDI regions such as Sub-Saharan Africa and Central Asia. The Slope Index of Inequality (SII) for ASPR narrowed from -2.597 to -1.087, reflecting reduced but persistent disparity. Rising ASMR and ASDR in high SDI countries like Germany and Norway underscore the need for targeted interventions. The negative SDI health correlation highlights socioeconomic influences. Prevention should focus on females in low-SDI areas, while improved medical care is needed for males facing higher mortality. Although a continued decline is projected, sustained efforts are essential in high burden countries. These findings, supported by a improving concentration index (CI) for ASPR (-0.358 to -0.218), reveal critical health inequalities and inform public health strategies.

## Author summary

We studied the impact of cystic echinococcosis (CE), a serious parasitic disease commonly acquired by dogs or livestock, on children and adolescents (0–19 years of age) globally from 1990 to 2021, and what may happen in the future. Using global Burden of disease data, we found that although the overall global burden of cystic echinococcosis has declined over these 32 years, major inequalities persist. Children and adolescents in areas with lower levels of socioeconomic development continue to bear a heavy burden, with consistently high incidence and mortality of CE. Infection rates are generally higher in girls than in boys, who are at higher risk of death. While our model predicts that the burden of disease will continue to decline slowly over the next 25 years, the disease is likely to remain a serious problem in some countries. Our findings highlight that cystic echinococcosis still poses a significant health threat to young people, especially in poorer areas. This study provides critical evidence to help global and national health leaders use resources and interventions more effectively to protect vulnerable children and adolescents, in line with the World Health Organization’s goal of controlling neglected diseases such as cystic echinococcosis by 2030.

## Introduction

Echinococcosis is a parasitic disease of zoonotic origin caused by tapeworms from the genus Echinococcus, epitomizing a significant example of neglected tropical diseases (NTDs). This disease is characterized by its intricate transmission ecology and its disproportionate health impacts on vulnerable populations [1–3]. Taxonomically, echinococcosis is primarily classified into three clinically distinct entities: Cystic Echinococcosis (CE), predominantly caused by ***Echinococcus granulosus*** sensu lato; Alveolar Echinococcosis (AE), resulting from ***Echinococcus multilocularis***; and the rare Polycystic forms attributed to ***E. vogeli*** and ***E. oligarthrus***, which are endemic to neotropical regions [4,5].

The larval metacestode stages preferentially colonize hepatic (60–70% of CE cases) and pulmonary tissues (20–30%), though disseminated infections may involve cerebral, osseous, and other organ systems, culminating in progressive morbidity and mortality in the absence of timely intervention [6,7]. CE is widely distributed globally, posing a significant threat to human health, especially in pastoral and rural communities [8]. Transmission dynamics are intrinsically linked to human-livestock-wildlife interfaces, with definitive hosts (canids) shedding infective eggs through fecal contamination of soil, water, and agricultural products [9–11]. Adolescents in pastoral communities exhibit heightened exposure risks due to behaviors such as herding, consumption of uncultivated vegetation, and limited access to sanitation infrastructure. Notably, pediatric populations demonstrate accelerated CE growth rates due to immunological naivety and developing organ systems, resulting in earlier clinical presentations compared to adults [12,13].

The diagnosis and treatment of echinococcosis entail considerable financial investment and often entail prolonged medical intervention [14,15]. Beyond the severe health implications, the disease exerts substantial socioeconomic repercussions, particularly resource-limited regions, presenting a considerable burden [16]. In certain endemic areas of CE, such as Central Asia, Eastern Europe, and specific pastoral areas of China, progress in reducing Disability-Adjusted Life Years (DALYs) remains sluggish or negligible. These regions typically face suboptimal medical conditions, inadequate prevention measures and insufficient medical resources, resulting in a persistent disease burden [17,18]. Despite the implementation of various control strategies across countries and regions, CE continues to pose a serious public health threat [9]. Therefore, a comprehensive understanding of the global disease burden of CE, especially in high-risk areas, is critical for informing public health initiatives.

The socioeconomic ramifications of CE are catastrophic in resource-constrained settings [16,19]. Surgical intervention, often combined with prolonged albendazole therapy, remains the standard for complicated CE, incurring costs two to three times the annual per capita health expenditure in low-income countries [7,20]. The management of CE requires long - term antiparasitic medication. Though surgery is the main treatment, it has a high recurrence rate, and surgical opportunities are scarce in resource - limited areas [21,22]. In 2019, there were an estimated 207,368 new cases of CE globally (95% UI = 137,807 – 303,233). In endemic regions, the annual age-standardized incidence rate (ASIR) of CE is between <1–200 per 100,000 people, with a age-standardized mortality rate (ASMR) of 2% – 4%. The global burden of CE in 2019 was on average 285,500 DALYs (over 1 million DALYs if underreporting is considered) [1,17,23]. Despite coordinated One Health initiatives, progress remains heterogenous; for example, western China achieved a 40% reduction in CE prevalence from 2016 to 2020 through dog deworming and livestock vaccination, whereas Central Asian republics report stagnant DALYs due to veterinary service fragmentation [24,25].

The risks associated with echinococcosis are essential for both travelers and healthcare providers to understand. Travelers visiting endemic regions may be at risk of infection through exposure to contaminated environments, particularly in rural areas [26]. For instance, contact with dog feces, consumption of untreated water, or ingestion of unwashed vegetables can lead to infection. Activities such as camping, hiking, or interacting with dogs in pastoral areas further increase exposure risks. Additionally, the long latency period of echinococcosis means infected individuals may remain asymptomatic for years, potentially delaying diagnosis and treatment [27,28]. Health professionals must equip travelers with preventive measures, including proper hygiene practices, such as handwashing after contact with animals or soil, and awareness of potential exposure routes, such as avoiding untreated water sources [29]. Furthermore, monitoring and managing cases of echinococcosis in travelers returning from endemic areas is crucial for public health surveillance [30]. Early detection and reporting of suspected cases, along with educating travelers about the symptoms and risks of echinococcosis, are vital steps in preventing the spread of the disease and ensuring timely medical intervention.

The World Health Organization (WHO) has established a roadmap for addressing neglected tropical diseases NTDs from 2021 to 2030, setting objectives aimed at enhancing control measures in 17 highly endemic countries by 2030, with CE singled out as a disease necessitating intensified intervention [2]. This initiative encourages international cooperation and underscores the necessity of fortifying control measures in high-incidence regions, aiming for comprehensive prevention and control [31]. In alignment with this WHO NTD Roadmap. we aim to study the critical evidence gaps regarding adolescent-specific CE burden.

The Global Burden of Disease (GBD) database is a crucial data platform offering in - depth insights into the global disease, injury, and risk factor burden. Although it covers many important diseases, current CE - related studies using GBD data mainly focus on overall disease burden assessment and trend forecasting, with relatively few targeting adolescents [32,33]. For instance, one study used the GBD 2019 database to analyze China’s CE disease burden from 1990 - 2019 and forecast the 2020–2044 trends, assessing the disease burden among various age groups through metrics such as ASIR, age-standardized prevalence rate (ASPR), ASMR and age-standardized DALY rate (ASDR) [1,25,34].

However, more in - depth research is needed on adolescent CE. Future studies could explore the epidemiological characteristics, disease burden, and control strategies specific to adolescents to better protect their health [35]. Systematically analyzing the echinococcosis data within the GBD database can clarify the disease’s geographical distribution, temporal changes, and demographic features, providing a scientific basis for effective control strategies. A thorough analysis of the echinococcosis burden at the global, regional, and national levels will enhance our understanding of the epidemiological trends and geographical inequities associated with the disease [25,36].

## Methods

### 1. Data sources

Data for this study were derived from the GBD 2021 database (https://ghdx.healthdata.org/gbd-results-tool), encompassing information related to CE across all age groups from 1990 to 2021. Through a comprehensive analytical framework and research design, our analysis specifically targeted the pediatric populations aged 0–19 years (<5, 5–9, 10–14,15–19 age groups). Key indicators extracted included prevalence, incidence, deaths, DALYs, Years Lived with Disability (YLDs), and Years of Life Lost (YLLs) due to CE. The GBD framework categorizes countries and regions into seven super regions and delineates 204 countries into 21 distinct GBD regions [36,37]. The Socio-demographic Index (SDI) serves as a composite metric reflecting the socio-economic development level of countries or regions [37]. The GBD classifies these countries and regions into five SDI quintiles: low SDI, low-middle SDI, middle SDI, high-middle SDI, and high SDI regions, allowing for a comprehensive exploration of health disparities. As this study is a secondary analysis of GBD data, we utilized pre-processed data directly from the GBD 2021 database, where all data processing procedures (including handling of missing data) were conducted by the GBD consortium. Detailed information on the GBD 2021 data processing pipeline, including methods for addressing missing data, can be found in the original GBD 2021 methodology publications [36,38].

### 2. Data processing

The burden of CE was quantified via various indicators across the designated pediatric age groups. To facilitate comparisons across years and regions, we calculated age-standardized rates (ASRs) per 100,000 population for the 0–19 age group. This process involved extracting the 0–19 age group population from the GBD 2021 standard reference population. We recalculated the new standard population composition ratio for the 0–19 subgroup and multiplied the crude rates of each 5-year age group within 0–19 years by their respective standard population weights [39]. The sum of these products yielded the ASRs per 100,000 population for the 0–19 age group [40].

The selection of the 0–19 age group as the focus for ASR calculation was driven by two key findings from our results: 1.Disproportionate disease burden in pediatric populations: Our analysis revealed that children and adolescents bear a significant CE burden relative to other age groups. For example, DALYs, YLDs, and YLLs—metrics reflecting health loss—were consistently higher in younger demographics (0–19 years) compared to older age groups. This pattern underscores the need to prioritize this population in burden assessments. 2.Unique epidemiological vulnerability: Pediatric populations exhibit distinct risk factors for CE, such as increased exposure to contaminated environments and immunological naivety, which accelerate cyst growth and lead to earlier clinical presentation. By focusing on 0–19 years, we aimed to capture these age-specific dynamics, which are critical for targeted public health interventions. Together, these factors justified the use of the 0–19 age group for ASR calculation, ensuring that our analysis accurately reflects the burden in a population uniquely affected by CE.

### 3. Statistical analysis

We analyzed the estimated annual percentage changes (EAPC) of the above six indicators to evaluate trends of pediatric echinococcosis burden. EAPC was calculated from regression coefficients, providing a standardized measure of disease burden changes. Subgroup analyses by age and region revealed trend variations.

Joinpoint regression, a statistical technique for identifying trend changes in time-series data, was used to analyze global ASRs of CE from 1990 to 2021 [23]. The analysis was performed using the Joinpoint Regression Program (version 5.2.0; National Cancer Institute, USA). The optimal number of joinpoints was determined by the Monte Carlo permutation test, with the number of potential joinpoints set between 0 and 5 [41]. In our analysis, we employed weighted log rate regression using inverse variance of rates to account for potential heteroscedasticity, maintaining the validity and reliability of the outputs. Trends were quantified using annual percent change (APC) and average annual percent change (AAPC). APC was calculated as (e^β - 1) × 100% (β being the regression coefficient of the logarithmic linear model), and AAPC as the weighted average of segment APCs with segment spans as weights. Both APC and AAPC were reported with 95% confidence intervals to enhance reporting accuracy. A trend was considered statistically significant if the 95% confidence interval did not include zero [37,40].

Health inequalities are defined as “unfair and avoidable differences in health across the entire population and between different social groups.” The Slope Index of Inequality (SII) and the Concentration Index (CI) are commonly used indicators to assess health inequality [42]. The detailed computational methodologies for these indicators are documented in the “Handbook on health inequality monitoring with a special focus on low- and middle-income countries” available on the WHO official website (https://www.who.int/publications/i/item/9789241548632). In our analysis, these indicators are employed to evaluate the differences in the ASRs of CE among groups defined by the different SDI levels of 204 countries. All analyses were population-weighted, using the country population size for each year as the weight. The SII quantifies the absolute difference in the ASR between the most and least socioeconomically advantaged countries, estimated via weighted linear regression. It is calculated as the regression coefficient ($\beta_{\text{1}}$) from the model:


$$y_{i}=\beta_{\text{0}}+\beta_{\text{1}}x_{i}+\epsilon_{i}$$

where $y_{i}$ is the ASR for country i, $x_{i}$ is its fractional SDI rank (ranging from 0 for the lowest to 1 for the highest SDI quintile), and the regression is weighted by the country’s population size ($\omega_{i}$). The CI quantifies relative socioeconomic inequality using the weighted covariance between the outcome ($y_{i}$) and the fractional SDI rank ($x_{i}$), calculated as:


$$CI=\frac{\text{2}}{\mu }\cdot \frac{\sum_{i=\text{1}}^{n}\omega_{i}\left(x_{i}-{\overset{―}{x}}_{\omega }\right)\left(y_{i}-{\overset{―}{y}}_{\omega }\right)}{\sum_{i=\text{1}}^{n}\omega_{i}}$$

where μ is the population-weighted mean of the ASR, ${\overset{―}{x}}_{\omega }$ and ${\overset{―}{y}}_{\omega }$ are the population-weighted means of the fractional rank and ASR respectively, and $\omega_{i}$ is the country’s population weight (S1 Text) [43,44]. The CI value ranges from -1–1, with negative values indicating a concentration of the health outcome among lower-SDI groups and positive values indicating concentration among higher-SDI groups. For our analysis, the CI was calculated using the inequal package in StataMP 17.

Correlation Analysis: To assess the association between the ASR of CE and the SDI, we performed a correlation analysis. Two methods were employed: the Pearson correlation coefficient was used to evaluate linear relationships, and Spearman’s rank correlation coefficient was used to assess monotonic relationships, as it is more robust to non-linearity and heteroscedasticity. This analysis was conducted using pooled global data from 204 countries and territories from 1990 to 2021 to assess the overall association trend; simultaneously, we also performed the analysis for each individual year to explore the temporal dynamics of the association. Non-linear relationships were visualized using Locally Weighted Scatterplot Smoothing (LOESS). Given that the primary objective of this analysis was to estimate the overall strength of the association, and considering the consistent effect sizes observed across years, no correction for multiple testing was applied. The focus of the analysis was on the effect sizes and their consistency.

Decomposition analysis was applied to evaluate the influence of population growth, aging, and epidemiological changes on CE burden (specifically, counts of and percentage changes) modifications from 1990 to 2021 based on the absolute number and ASRs of each age groups (0–4, 5–9, 10–14, 15–19 years). This statistical tool is valuable in epidemiological research for analyzing interactions among factors affecting health outcomes [44,45]. The decomposition was performed following the robust method developed by Cheng et al. (2020). This approach provides a unique and determinate solution, explicitly decomposing the change in the number of cases into the independent contributions of: 1) population size change, 2) population age structure change (aging), and 3) change in age-specific incidence rates (epidemiologic change). The exact formulas used, corresponding to equations (1) to (10) in Cheng et al. (2020) [46].

Frontier analysis is a reliable methodological tool for evaluating and boosting healthcare systems efficiency through benchmarking against top-performing entities [32]. This analysis was conducted to quantitatively evaluate the relationship between the CE burden and socio-demographic development, utilizing ASDR and SDI data spanning from 1990 to 2021. We employed non-parametric Data Envelopment Analysis (DEA) to establish a nonlinear frontier that represents the minimum achievable burden based on the SDI level [41,47]. We specified an output-oriented model under variable returns to scale (VRS) to reflect the realistic operational scale of health systems and to model the potential reduction of an undesirable output (ASDR) for a given level of SDI. To account for stochasticity and sampling variability inherent in the data, we implemented a bootstrapping procedure with 1000 repetitions. This bootstrap was used to construct bias-corrected confidence intervals for the frontier itself, allowing for a more robust identification of outliers (i.e., countries with an ASDR significantly above the expected minimum). The input (SDI) and output (ASDR) data were year-matched but not smoothed, as we aimed to capture the actual annual relationship. DEA was chosen over quantile regression (e.g., for the 5th percentile) because it is non-parametric and makes fewer distributional assumptions, it directly constructs a piece-wise linear frontier from the best-performing units, providing a clearer benchmark for “best achievable” performance rather than a statistical expectation.

The Bayesian Age-Period-Cohort (BAPC) model, leveraging the BAPC package in R, was employed to predict the ASRs of CE among the 0–19 age group. We confirm that our model specification utilized a Poisson likelihood with a log link function and the logarithm of the population as an offset. This is an intrinsic default setting of the BAPC function from the R BAPC package. We modeled the count of cases using a Poisson likelihood with a log-link function. An offset term, defined as the log of the corresponding age-specific population, was included to convert the modeled counts into rates. The model was constructed within the Integrated Nested Laplace Approximations (INLA) approach. This framework enables a comprehensive assessment of age, period, and cohort effects. For the age, period, and cohort effects, we assigned them the commonly used second-order random walk (RW2) prior distributions. For the precision parameters, we adopted a log-gamma prior distribution with shape = 1 and scale = 0.00005, a relatively uninformative prior chosen to allow data to dominate the inference. The sensitivity of results to this prior choice was assessed by comparing with alternative hyperparameters, which yielded consistent posterior estimates. The posterior characteristics of these precision hyperparameters are provided in S1 Table. To ensure the comparability of results, we standardized the weights. We set secondDiff = FALSE to preserve the original trends of the model and retro = TRUE to generate predictions for historical data. The demographic structure from the GBD2021 standardization population was used to normalize the results. To assess the calibration and accuracy of our forecasts to 2030 and 2050, we performed a temporal hold-out validation by fitting the model on data from 1990 to 2010 and forecasting the years 2011–2021. The forecasts were evaluated using the predication comparison, relative prediction difference and correlation comparisons. Posterior predictive checks indicated that the model adequately captured the central tendency of the observed data. The wide 95% prediction intervals for the forecasted years, particularly for YLDs, are closely associated with data sparsity and heterogeneity. The Joinpoint results revealed that the trends of prevalence, incidence, and YLDs exhibited irregular fluctuations over the years, which might introduce considerable noise and interference to the subsequent analyses. In certain periods or age groups, the number of observations was relatively small, reflect the substantial heterogeneity in the data across regions and the inherent uncertainty in long-term projections. Our projections represent the expected outcomes under the scenario of no subsequent interventions. However, due to uncertainties from various factors (such as changes in population dynamics, evolving environmental conditions, and potential shifts in disease transmission patterns, etc.), there is a certain probability that the final actual results may not fall within the predicted intervals. By integrating Bayesian statistics with the age - period - cohort framework, the BAPC model provides a powerful tool for disease burden forecasting [1,48]. The complete R code used for the analysis, along with a description of the input data structure, is provided in the Supplementary Material to ensure full reproducibility. (S2 Text).

The data analysis for this study was conducted from October 30, 2024, to December 1, 2024. All analyses were performed using R version 4.3.3 and StataMP 17. The reporting of this work adheres to the corresponding reporting standards.

## Results

### 1. Global variations analyzed

**Fig 1:**
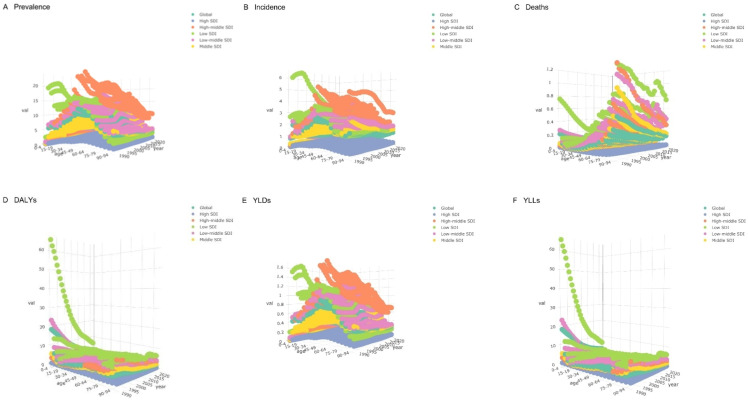
Distribution of ASRs of childhood and adolescent CE across Global and five SDI regions and age groups. **(A)** Prevalence; **(B)** Incidence; **(C)** Deaths; **(D)** DALYs; **(E)** YLDs; **(F)** YLLs.

Our analysis firstly elucidates the variations in the disease burden of CE across different age groups, years, and SDI levels globally. Specifically, it was observed that the ASPR varies significantly across regions with differing SDI levels; high SDI areas exhibit lower ASPR, whereas low SDI areas show higher ASPR. The ASIR is notably higher among younger populations and gradually decreases with increasing age. Regions with high SDI have lower ASIR compared to low SDI regions. ASMR exhibits substantial differences across SDI levels, with a marked increase in populations aged 75 and above in low SDI areas. DALYs, YLDs, and YLLs are higher among young demographics and tend to decrease with age. Given the elevated metrics among children and adolescents, we have chosen to focus our study on these age groups as the primary subjects of this investigation (Fig 1).

### 2. Descriptive analysis

#### 2.1 Global level.

In 2021, the global prevalence of CE among children and adolescents was estimated at 101,104.81 cases (95% UI: 59,547.85-160,779.33), yielding an ASPR of 3.71 (95% UI: 2.18-5.9). The global incidence was recorded at 30,135.07 cases (95% UI: 16,309.43-50,135.21), corresponding to an ASIR of 1.12 (95% UI: 0.61-1.86). The global number of deaths attributable to CE was 289.98 cases (95% UI: 63.86-626.07), resulting in an ASMR of 0.01 (95% UI: 0-0.02). Additionally, the global DALYs for CE among children and adolescents was estimated at 31,665.29 (95% UI: 11,033.01-59,829.13), with an ASDR of 1.23 (95% UI: 0.43-2.33). From 1990 to 2021, a notable declining trend was observed in global ASPR (EAPC = -0.91, 95% UI: -1.03 to -0.79), ASIR (EAPC = -0.76, 95% UI: -0.88 to -0.64), ASMR (EAPC = -6.31, 95% UI: -6.45 to -6.17), and ASDR (EAPC = -5.78, 95% UI: -5.88 to -5.68) (Table 1).

**Table 1:** Core CE burden indicators among children and adolescents by global and SDI quintiles (2021 and 1990-2021 Trends).

Location	Prevalence			Incidence			Deaths			DALYs		
	Prevalence cases in 2021 (95%UI)	ASPR in 2021 (95%UI)	EAPC from 1990 to 2021 (95%CI)	Incidence cases in 2021 (95%UI)	ASIR in 2021 (95%UI)	EAPC from 1990 to 2021 (95%CI)	Deaths cases in 2021 (95%UI)	ASMR in 2021 (95%UI)	EAPC from 1990 to 2021 (95%CI)	DALYs cases in 2021 (95%UI)	ASDR in 2021 (95%UI)	EAPC from 1990 to 2021 (95%CI)
**Global**	**101104.81(59547.85-160779.33)**	**3.71(2.18-5.9)**	**-0.91(-1.03--0.79)**	**30135.07(16309.43-50135.21)**	**1.12(0.61-1.86)**	**-0.76(-0.88--0.64)**	**289.98(63.86-626.07)**	**0.01(0-0.02)**	**-6.31(-6.45--6.17)**	**31665.29(11033.01-59829.13)**	**1.23(0.43-2.33)**	**-5.78(-5.88--5.68)**
High SDI	1349.29(710.75-2315.07)	0.53(0.28-0.91)	-0.08(-0.17-0.01)	427.53(211.6-762.12)	0.17(0.08-0.31)	-0.05(-0.15-0.06)	0.46(0.01-1.39)	0(0-0)	-7.85(-7.94--7.77)	135.74(56.64-250.88)	0.05(0.02-0.1)	-4.73(-4.97--4.49)
High-middle SDI	12180.4(7184.63-19505.12)	3.79(2.24-6.07)	0.14(-0.01-0.29)	3817.28(2036.91-6407.23)	1.2(0.64-2.01)	0.13(-0.05-0.3)	5.38(0.38-14.53)	0(0-0)	-8.03(-8.39--7.66)	1324.73(571.05-2392.25)	0.42(0.18-0.77)	-5.39(-5.54--5.24)
Middle SDI	22714.72(12963.99-36686.04)	2.86(1.63-4.62)	0.28(0.17-0.4)	7066.98(3708.16-11994.98)	0.9(0.47-1.53)	0.48(0.35-0.61)	21.9(1.18-59.97)	0(0-0.01)	-7.33(-7.49--7.17)	3434.63(1135.14-6887.02)	0.45(0.14-0.92)	-5.9(-5.96--5.84)
Low-middle SDI	27585.73(15857.97-44458.09)	3.47(2-5.6)	-1.77(-1.87--1.66)	8073.6(4276.93-13405.79)	1.03(0.55-1.72)	-1.48(-1.57--1.38)	81.86(12.86-183.48)	0.01(0-0.02)	-7.25(-7.39--7.1)	8769.76(2574.5-17221.25)	1.16(0.34-2.29)	-6.77(-6.86--6.68)
Low SDI	37210.15(21980.96-59151.2)	6.48(3.83-10.31)	-2.75(-2.93--2.58)	10730.44(5911.25-17768.76)	1.85(1.02-3.07)	-2.49(-2.64--2.33)	180.25(48.43-367.34)	0.03(0.01-0.06)	-6.99(-7.09--6.88)	17984.36(6273-33628.67)	3.02(1.05-5.65)	-6.66(-6.76--6.57)

Note: ASPR, ASIR, ASMR, ASDR (per 100,000 population)

#### 2.2 Regional level.

At the SDI regional level, disparities were observed in the levels and trends of ASPR for children and adolescents across different SDI regions in 2021. The ASPR was highest in low SDI regions, reaching 6.48 (95% UI: 3.83-10.31), while it was lowest in high SDI regions, recorded at 0.53 (95% UI: 0.28-0.91). The EAPC of ASPR indicated a notable decline trend, particularly in low SDI regions, with an EAPC of -2.75 (95% UI: -2.93--2.58), indicating relatively more substantial improvement in this region. In terms of ASMR, the highest rate was also found in low SDI regions, at 0.03 (95% UI: 0.01-0.06), followed by low-middle SDI regions, at 0.01 (95% UI: 0-0.02). From 1990 to 2021, the overall trend of ASMR showed a decrease, with the annual change sequence from high to low being: low SDI regions, low-middle SDI regions, middle SDI regions, high SDI regions, and high-middle SDI regions. Similarly, the annual change in ASDR also exhibited a decreasing trend, with the change sequence from high to low being: high SDI regions, high-middle SDI regions, middle SDI regions, low SDI regions, and low-middle SDI regions (Table 1).

At the level of seven super-regions, the rankings of ASPR and ASIR in 2021 were consistent, with the following order: North Africa and the Middle East, Sub-Saharan Africa, Central Europe, Eastern Europe and Central Asia, South Asia, High-income regions, Southeast Asia, East Asia and Oceania, and Latin America and the Caribbean. Conversely, the ranking order for ASMR and ASDR in 2021 was led by Sub-Saharan Africa, followed by South Asia, North Africa and the Middle East, Southeast Asia, East Asia and Oceania, Central Europe, Eastern Europe and Central Asia, Latin America and the Caribbean, and High-income regions. From 1990 to 2021, all super-regions, with the exception of Central Europe, Eastern Europe and Central Asia, Southeast Asia, East Asia and Oceania (which demonstrated an EAPC > 0), exhibited a negative growth in ASPR and ASIR (EAPC < 0). Over the past 32 years, both ASMR and ASDR have consistently shown a declining trend. (S2 Table)

At the level of 21 GBD regions, South Latin America in high-income regions exhibited the highest ASPR and ASIR, followed by East Sub-Saharan Africa in Sub-Saharan Africa, which recorded the highest ASMR and ASDR. From 1990 to 2021, except for Australasia, High-income North America, East Asia, Oceania, and Central Asia (ASIR with EAPC > 0), other regions showed negative growth in ASPR and ASIR (EAPC < 0). Throughout the past 32 years, a majority of GBD regions displayed a declining trend in ASPR, ASIR, ASMR, and ASDR. Notably, East Asia experienced the most pronounced increasing trend in ASPR (4.92, 95% UI: 4.13-5.72) and ASIR (5.15, 95% UI: 4.34-5.97), but also faced the most significant decrease in ASMR (-10.68, 95% UI: -11.06 to -10.29). Meanwhile, Andean Latin America exhibited the most significant decreasing trend in ASDR (-7.15, 95% UI: -7.42 to -6.89) (S3 Table).

#### 2.3 National level.

**Fig 2:**
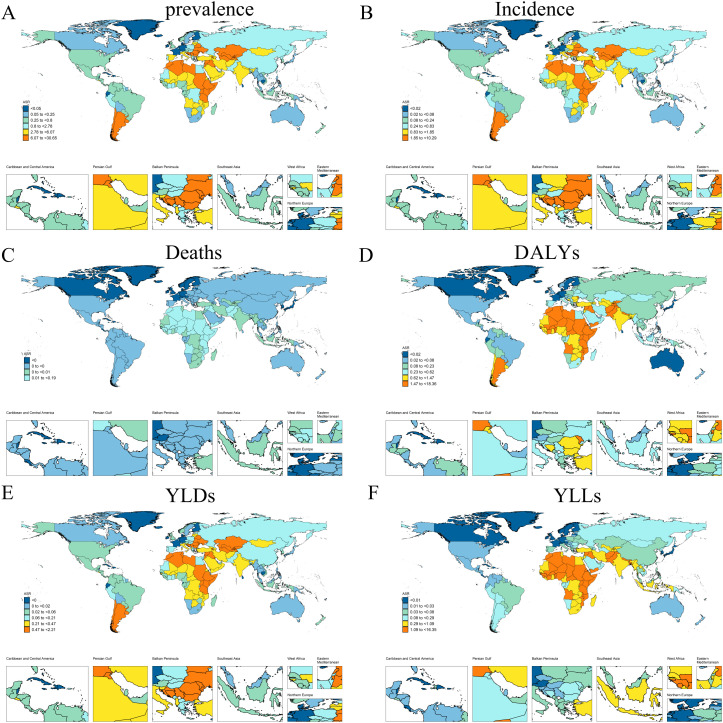
Map of ASRs of childhood and adolescent CE across 204 countries in 2021 (https://www.resdc.cn/). **(A)** Prevalence; **(B)** Incidence; **(C)** Deaths; **(D)** DALYs; **(E)** YLDs; **(F)** YLLs.

In 2021, there was a marked variation in the ASPR of CE among children and adolescents globally across different countries. Moldova reported the highest ASPR at 30.65 (95% UI: 17.62-48.48) and an ASIR of 10.29 (95% UI: 5.53-17.15). Conversely, Iceland exhibited the lowest ASPR at 0.04 (95% UI: 0.01-0.09), while Greenland, Monaco, and San Marino recorded the lowest ASIR at 0.01 (95% UI: 0-0.03). From 1990 to 2021, China experienced significant increases in ASPR (EAPC = 5.04%, 95% CI: 4.23-5.86) and ASIR (EAPC = 5.27%, 95% CI: 4.43-6.11). Conversely, Liberia showed the most pronounced decline in ASPR (EAPC = -4.32%, 95% CI: -4.71--3.93) and ASIR (EAPC = -3.86%, 95% CI: -4.21--3.5). Notably, South Sudan reported the highest ASMR at 0.19 (95% UI: 0.08-0.36), while Ethiopia recorded the highest ASDR at 8.42 (95% UI: 3.67-14.37). Zimbabwe demonstrated the most significant increasing trend in ASDR (EAPC = 1.09%, 95% CI: -0.01-2.2), whereas Equatorial Guinea experienced the most substantial decrease in ASDR (EAPC = -11.38, 95% CI: -11.96--10.8) (Fig 2 and S3 Table).

### 3. Analysis of gender differences

**Fig 3:**
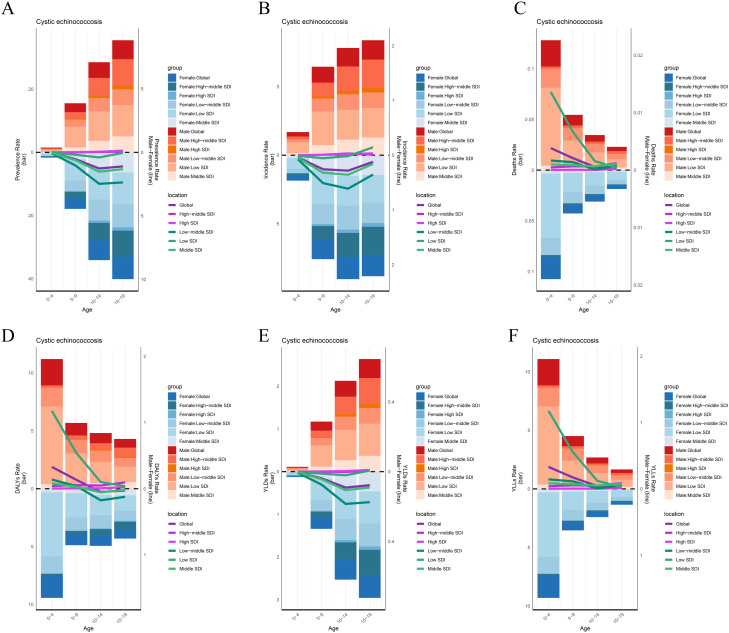
ASRs of CE in children and adolescents worldwide by gender and age group across global and SDI Regions in 2021. (The lines in the figure denote the subtraction of female ASRs from male ASRs for each age group in different SDI locations.) **(A)** Prevalence; **(B)** Incidence; **(C)** Deaths; **(D)** DALYs; **(E)** YLDs; **(F)** YLLs.

We provide a comprehensive examination of the epidemiological characteristics of CE in children and adolescents on a global scale, encompassing key indicators including prevalence, incidence, mortality, DALYs, YLDs, and YLLs. The analysis revealed that prevalence tends to increase with age across most SDI regions, peaking in the 15–19 age group (Fig 3A). Notably, in regions other than those with high SDI levels, females generally have a higher prevalence than males, particularly in low- to middle-SDI regions (Fig 3A). Similar trends are observed in ASIR, with the most significant gender differences occurring in the 10–14 age group (Fig 3B). ASMR are highest in the 0–4 age group, with a gradual decline observed as age increases; males consistently demonstrate higher ASMR compared to females across all age groups (Fig 3C). Concurrently, both DALYs and YLLs reflect a similar trend to that of mortality, peaking in the 0–4 age group before declining with age (Fig 3D and 3F). In contrast, YLDs show a pattern similar to prevalence (Fig 3E). Gender differences are evident across all health indicators (Fig 3). Females bear a greater burden in terms of prevalence and incidence, whereas males bear a greater burden regarding mortality. In high SDI regions, all disease burden indicators demonstrate improved performance (lower rates), with relatively smaller gender differences observed (Fig 3).

### 4. Joinpoint trend analysis

**Fig 4:**
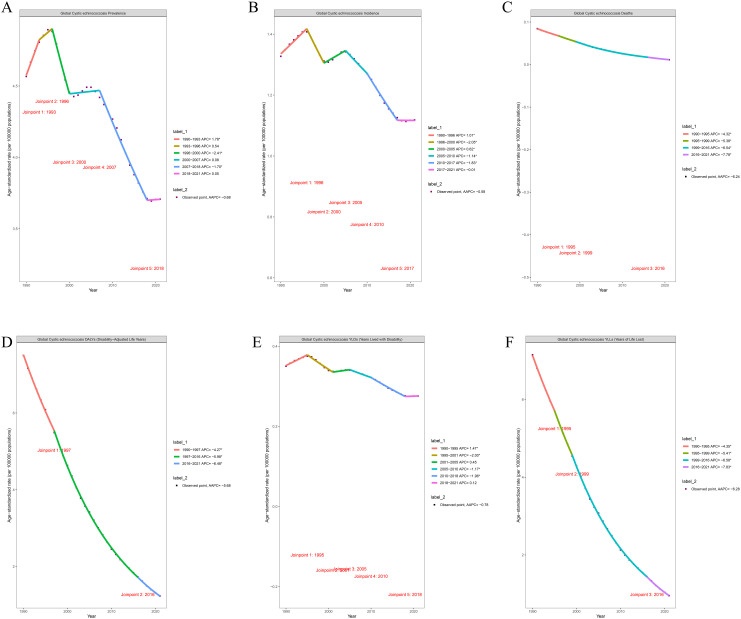
Joinpoint regression analysis of age-standardized global CE burden in children and adolescents (1990–2021). **(A)** Prevalence; **(B)** Incidence; **(C)** Deaths; **(D)** DALYs; **(E)** YLDs; **(F)** YLLs.

Joinpoint regression analysis of the global ASR for CE indicates a general decline in both the ASPR and ASIR among children and adolescents worldwide from 1990 to 2021. The AAPC for ASPR and ASIR was -0.68% and -0.58%, respectively (Fig 4A and 4B). Similarly, the ASMR and ASDR have exhibited a downward trend, with AAPCs of -6.24% and -5.68%, respectively (Fig 4C and 4D). It is noteworthy that between 1990 and 1996, the ASPR with APC of 1.78% from 1990 to 1993 and 0.54% from 1993 to 1996) and the ASIR (APC of 1.01%) showed an increasing trend. Over the entire period from 1990 to 2021, the prevalence, incidence, mortality, DALYs, YLDs, and YLLs for CE in children and adolescents globally have all demonstrated a downward trend (Fig 4E and 4F).

### 5. Health inequality analysis

**Fig 5:**
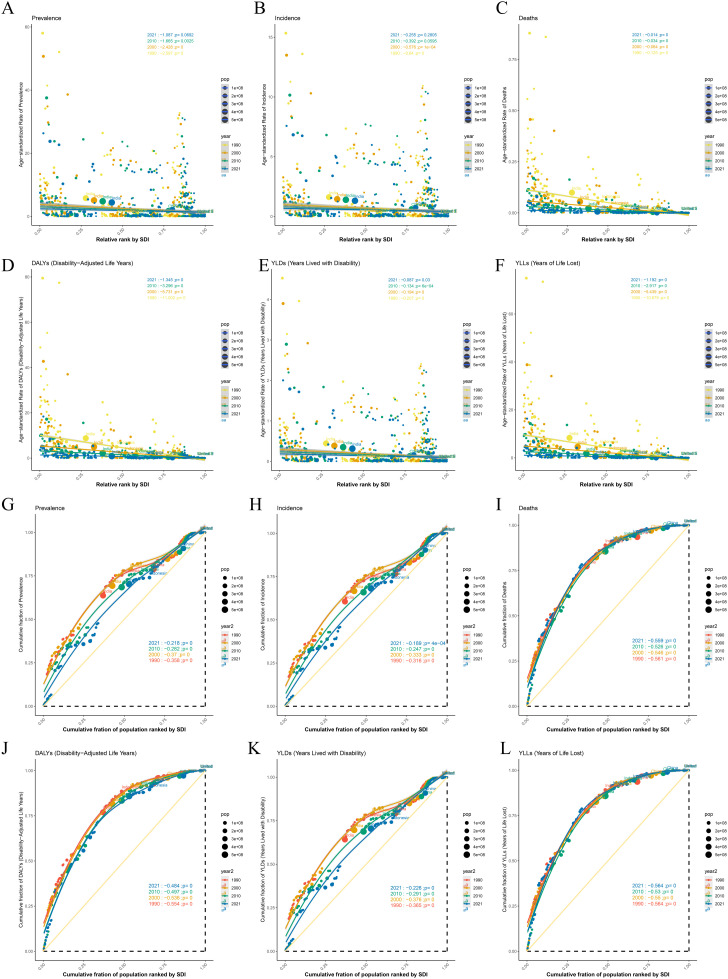
Global health inequality regression analysis for ASRs of CE in children and adolescents (1990 and 2021). **(A)** Slope index of Prevalence; **(B)** Slope index of Incidence; **(C)** Slope index of Deaths; **(D)** Slope index of DALYs; **(E)** Slope index of YLDs; **(F)** Slope index of YLLs. The concentration index Global concentration of global health inequality in age-standardized CE among children and adolescents in 1990 and 2021. **(G)** Concentration index of Prevalence; **(H)** Concentration index of Incidence; **(I)** Concentration index of Deaths; **(J)** Concentration index of DALYs; **(K)** Concentration index of YLDs; **(L)** Concentration index of YLLs.

Between 1990 and 2021, global health inequality analysis reveals significant improvements in various health indicators, particularly among children and adolescents. The SII for ASPR for childhood and adolescents CE decreased from -2.597 to -1.087, highlighting a narrowing gap between socioeconomic groups, especially in low SDI regions. ASPR dropped markedly, with decreases of 2.428 in 2000 (p < 0.0001), 1.665 in 2010 (p = 0.0025), and 1.087 in 2021 (p = 0.0092). Conversely, high SDI regions exhibited lower and more stable ASPR (Fig 5A).

The slope index for ASIR shifted from -0.64 to -0.255, demonstrating substantial decreases of 0.578 in 2000 (p < 0.0001), 0.392 in 2010 (p = 0.0596), and 0.255 in 2021 (p = 0.2805), indicating a deceleration in downward trends, particularly in low SDI areas(Fig 5B). ASMR also showed a decrease from -0.125 to 0.014, with significant annual reductions in 2000 (0.084, p = 0.0001), 2010 (0.034, p = 0.0001), and 2021 (0.014, p = 0.0001). While the rate of decline diminished, high SDI regions consistently reported lower ASMR (Fig 5C).

Furthermore, ASDR saw a significant decline from -11.002 to -1.345, with reductions in disease burden particularly pronounced in low SDI regions. YLDs and YLLs also significantly decreased, underscoring improvements in health outcomes for lower socioeconomic groups (Fig 5D,5E and 5F).

Additionally, concentration index data indicated a reduction in health inequity, with the ASPR index rising from -0.358 to -0.218, reflecting a less concentrated prevalence of adverse health outcomes among lower socioeconomic populations. The ASIR index rose from -0.316 to -0.189, and the ASDR index improved from -0.554 to -0.484, indicating notable progress in reducing health inequities (Fig 5G,5H and 5J).

Overall, from 1990 to 2021, health outcomes have improved across all SDI levels, particularly benefiting lower socioeconomic groups, thereby moderating the historical concentration of negative health outcomes in these populations.

### 6. The impact of SDI on the burden of CE diseases in children and adolescents

This study demonstrates a significant negative correlation between the SDI and ASRs of CE - related health indicators among children and adolescents, indicating a general decline in CE - related disease burdens as SDI increases.

**Fig 6:**
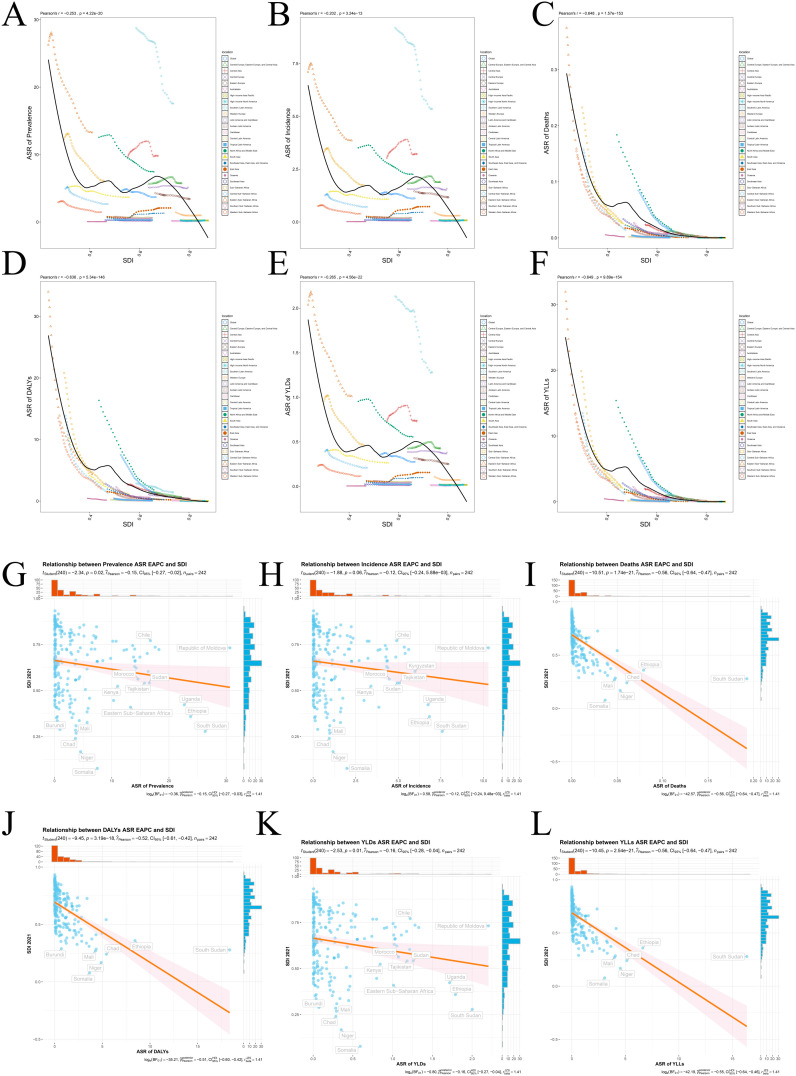
Correlation between SDI and ASRs of CE among children and adolescents). correlation in 1990- 2021 for CE among children and adolescents. Scatter plot of SDI and ASRs correlation in GBD locations (1990- 2021): **(A)** Prevalence; **(B)** Incidence; **(C)** Deaths; **(D)** DALYs; **(E)** YLDs; **(F)** YLLs. Correlation Analysis of SDI and ASRs in 204 countries for 2021: **(G)** Prevalence; **(H)** Incidence; **(I)** Deaths; **(J)** DALYs; **(K)** YLDs; **(L)** YLLs.

At the regional level, SDI shows negative correlations with CE prevalence (r = - 0.253, p < 0.0001), incidence (r = - 0.202, p < 0.0001), mortality (r = - 0.648, p = < 0.0001), DALYs (r = - 0.636, p = < 0.0001), YLDs (r = - 0.265, p = < 0.0001), and YLLs (r = - 0.649, p = < 0.0001) (Fig 6A,6B,6C,6D,6E and 6F). High - SDI countries have more pronounced downward trends in these health indicators, particularly in mortality and DALYs. At the national level, SDI remains significantly negatively correlated with ASRs of CE health indicators, including prevalence (r = - 0.15, p < 0.05), incidence (r = - 0.12), mortality (r = - 0.56, p < 0.0001), DALYs (r = - 0.52, p < 0.0001), YLDs (r = - 0.16, p < 0.05), and YLLs (r = - 0.56, p < 0.0001) (Fig 6G,6H,6I,6J,6K and 6L). Low - SDI countries like Chad, Somalia, and Niger have higher disease burdens, while high - SDI countries have lower burdens.

Data indicate a significant negative correlation between SDI and CE - related health indicators, a trend widely found across different countries and regions. Low - SDI countries generally have higher disease burdens, while high - SDI countries have lower burdens.

### 7. Epidemiological drivers decomposition analysis

**Fig 7:**
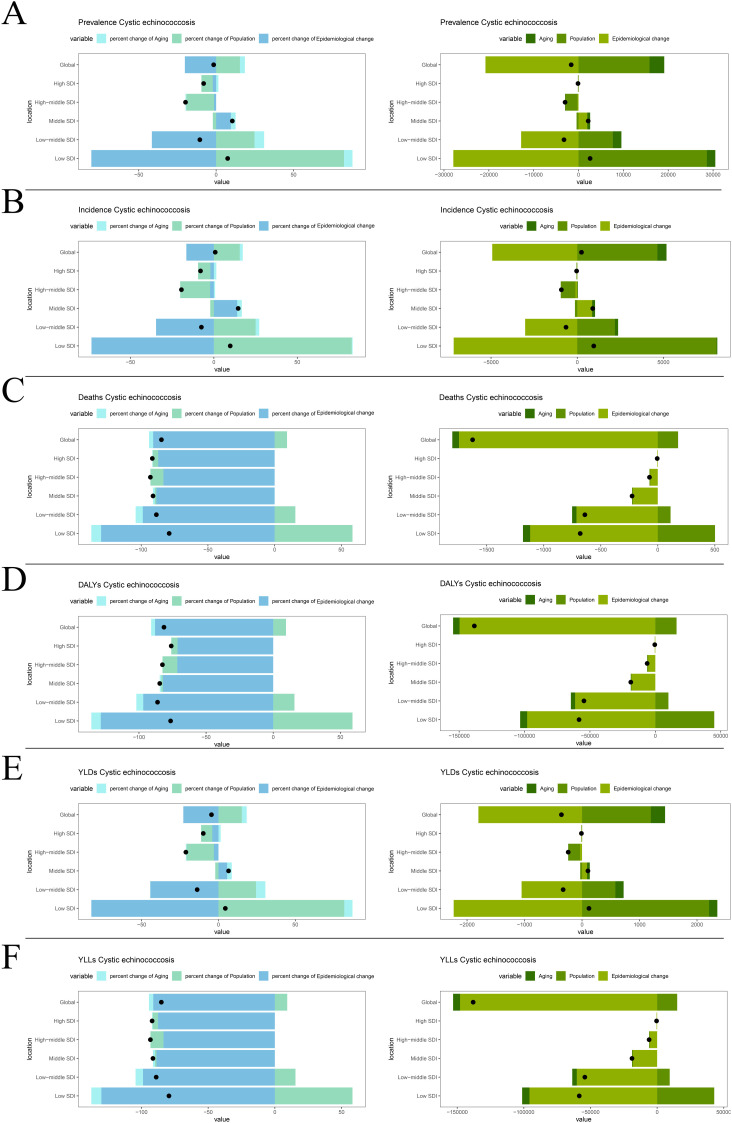
Decomposition analysis of CE burden among children and adolescents globally and across five SDI regions (1990 to 2021). (A) Prevalence; (B) Incidence; (C) Deaths; (D) DALYs; (E)YLDs; (F)YLLs.

To assess the impact of population growth, ageing, and epidemiological changes over the past 32 years on communicable diseases CE in children and adolescents, we performed a disaggregation analysis of epidemiological factors. The results showed that, globally, epidemiological changes became the dominant factor affecting the prevalence, contributing -20.2% to the global prevalence change. Epidemiological changes significantly affected prevalence in low-middle SDI regions, whereas the effect was -1.4% in high-middle SDI regions. Areas with low SDI were influenced by both demographic and epidemiological changes, with demographic factors accounting for 82.6% of the change in prevalence and epidemiological changes accounting for -80.6% (Fig 7A). Trends in incidence, similar to those in prevalence, were driven globally by major epidemiological changes, which accounted for -16.5% of the change in incidence. In middle and low-middle SDI regions, epidemiologic factors played a more important role, whereas high SDI regions showed more complex effects (Fig 7B). Changes in mortality were driven primarily by epidemiologic changes globally and in all SDI regions, accounting for -91.1% of the global change in mortality, whereas low SDI regions were significantly affected by epidemiologic changes (-130.1%) (Fig 7C). The change in DALYs reflects the combined effects of incidence, prevalence and mortality, and the change in DALYs was -88.0% influenced by epidemiological changes globally. Across all SDI regions, DALYs were also primarily driven by epidemiological changes, underscoring the importance of disease prevention measures (Fig 7D). Changes in YLDs and YLLs were influenced by epidemiological changes at the global scale by -22.9 and -91.2, respectively (Fig 7E and 7F).

### 8. Frontier analysis of mortality and DALY

An analysis of echinococcosis - related mortality and DALYs among children and adolescents aged 0–19 across 204 countries and regions from 1990 to 2021 revealed a correlation between these health indicators and the SDI, providing a basis for assessing health outcomes at different SDI levels. Further frontier analysis explored health status across various SDI levels. In the analysis, a boundary line (black curve) represented the ideal health status achievable by countries or regions under specific SDI conditions. This helped identify countries or regions underperforming relative to expectations, indicating key areas for public health improvement. Conversely, regions performing better than expected could be models, offering valuable experiences and strategies to others.

**Fig 8:**
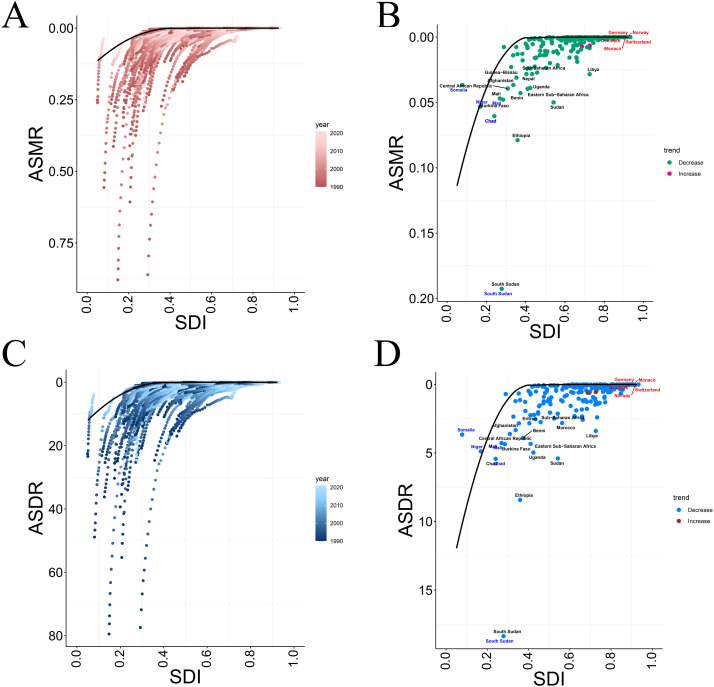
Frontier analysis of CE based on SDI and ASDR or ASMR among children and adolescents. **(A)** Frontier analysis results of ASMR from 1990 to 2021; **(B)** Frontier analysis of ASMR in 2021; **(C)** Frontier analysis results of ASDR from 1990 to 2021; **(D)** Frontier analysis of ASDR in 2021. (A-B) ASMR; **(C-D)** ASDR.

In - depth analysis of adolescent health indicators across 204 countries and regions focused on the relationship between SDI and ASMR & ASDR. Figs A and C show that ASMR and ASDR of echinococcosis among children and adolescents decreased with increasing SDI. The color gradient indicates that even in low - SDI regions, ASMR and ASDR have declined over time, reflecting improved global health and strengthened disease control (Fig 8A and 8C). Figs B and D detail the downward trends of ASMR and ASDR, which are at low levels in most countries, especially high - SDI ones like Germany, Norway, and Switzerland, consistent with the expected positive link between socio - economic development and health improvement (Fig 8B and 8D). However, some low - SDI countries, such as Niger and Somalia, still have high ASMR and ASDR, pointing to unique health - related challenges. Additionally, in Figs B and D, Germany, Norway, and Switzerland are marked in red, indicating an upward trend in ASDR and ASMR, contrasting with the downward trend in most countries. These countries’ situations need further analysis. Meanwhile, low - SDI countries like Niger, Chad, and Somalia, marked in blue, also show a downward trend in ASMR and ASDR despite their low SDI, signaling positive health improvements (Fig 8B and 8D).

The analysis highlights the differences in adolescent health among countries and points out that in some regions, especially those with lower SDI, the declining trend of ASMR and ASDR may not be obvious. Fig 8 visually demonstrates that countries closer to the ideal health status boundary line have better health status, while those farther away need improvement. Germany, Norway, and Switzerland are close to the line and perform well, while Sudan and Ethiopia are much farther away and perform poorly (Fig 8)

### 9. Web-APC analysis of CE burden in children and adolescents

**Fig 9:**
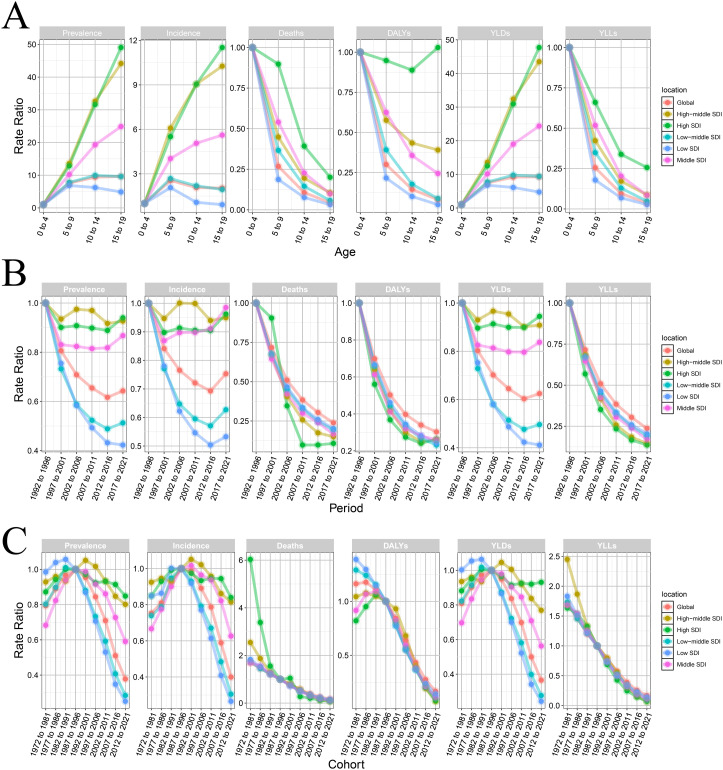
Estimating global age-period-cohort effects of CE among children and adolescents using Web Age-Period-Cohort (APC) analysis. (A) age effect; (B) period effect; (C) cohort effect.

We employs the Web-APC framework to investigate the disease burden of CE among children and adolescents across different SDI locations. Age-specific assessments indicated a notable increase in prevalence with advancing age, particularly pronounced in high and high-middle SDI regions. In contrast, low SDI regions exhibited a peak prevalence at ages 5–9 followed by a subsequent decline. The DALYs, YLDs, and YLLs metrics reflected a more substantial impact on quality of life and life expectancy in high SDI regions compared to lower SDI regions. A decreasing mortality trend was observed across all age groups, with high SDI regions relatively exhibiting relatively elevated prevalence, incidence, mortality, DALYs, YLDs, and YLLs (Fig 9A).

Period analysis spanning 1992–2021 showed declining trends in prevalence, incidence, and YLDs across all regions, with the most notably in low-income and lower-middle-income regions. High-income and high-middle-income regions demonstrated relative stability, characterized by fluctuations between 1997 and 2006, yet lacking any significant overall change. Mortality, DALYs, and YLLs decreased significantly across all regions, particularly in high-income areas (Fig 9B).

Cohort analysis revealed consistent relative risks for prevalence, incidence, and YLDs among individuals born between 1987 and 1996 with marked declines observed in low-income and lower-middle-income regions in subsequent cohorts. In contrast, high-middle-income regions initially exhibited an increase in relative risk, followed by a decrease in relative risk. From 1972 to 2021, ASMR alongside DALYs and YLLs, have generally declined across most regions (Fig 9C). Overall, the analysis underscores that advancements in public health intervention have contributed to significant reductions in prevalence, incidence, and mortality from CE.

### 10. Forecast analysis of echinococcosis burden in children and adolescents

**Fig 10:**
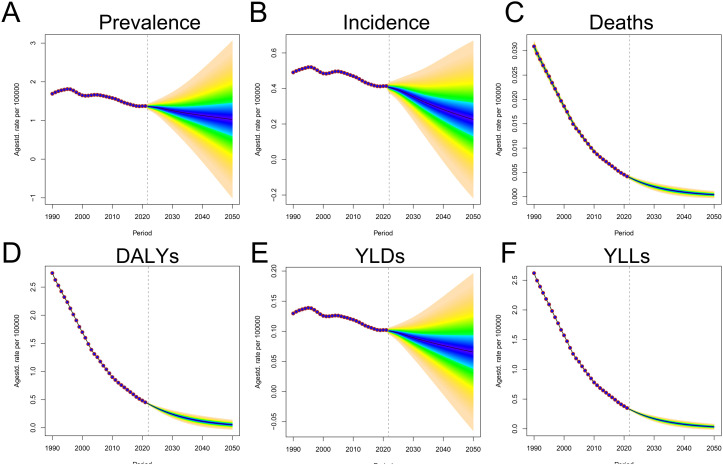
Global projection of CE burden among children and adolescents by 2050 using Bayesian Age-Period-Cohort (BAPC) modeling. (A) ASPR; (B) ASIR; (C) ASMR; (D) ASDR; (E) YLDs; (F) YLLs.

Predictive analyses on CE indicate promising trends in the global prevalence, incidence, and ASR of YLDs among children and adolescents with CE (Fig 10A,10B and 10E). While a continued decline in these measures is anticipated, the rate of decrease is expected to slow. The broad prediction intervals associated with these metrics introduce significant uncertainty surrounding their future projections. Conversely, the predictions for ASR related to mortality, DALYs, and YLLs indicate a sustained decline approaching zero, supported by narrow prediction intervals (Fig 10C,10D and 10F). This suggests a higher level of confidence in the future mortality data. It is anticipated that all measured indicators, particularly mortality, DALYs, and YLLs, will continue to decline by 2050, signifying a further reduction in the global CE burden. The validation analysis utilized data from 1990 to 2010 to predict metrics spanning 2011–2021 and then compared these forecasts with actual data from that period, yielded the following results: For the years 2020–2021, the ASRs were predominantly within the 95% CIs of the predicted ASRs. Specifically, the ASRs related to deaths, DALYs, and YLLs exhibited relatively high predictive accuracy, with the relative differences remaining within 12% even after a decade. In contrast, the predictions for prevalence, incidence, and YLDs were less precise, accompanied by wider 95% confidence intervals compared to those for death-related metrics. Notably, the relative mean difference ratio for incidence reached 22.4 ± 2.3%. Furthermore, there was a significant correlation between the forecasted values for 2011–2021 and the observed values, with all correlation coefficients (r) exceeding 0.93 (S3 Text). However, the wide prediction intervals for prevalence, incidence, and YLDs indicate considerable uncertainty regarding future changes in these specific outcomes. This uncertainty underscores the need for ongoing surveillance and research to better understand the factors influencing these trends and to refine predictive models accordingly. This way, we can take a proactive approach in public health planning and intervention strategies to face future challenges.

## Discussion

CE poses a significant threat to global human health and also presents challenges to animal husbandry [32]. In 2019, there were an estimated 207,368 new cases globally [1]. The peak incidence of the disease occurs in childhood. Due to a prolonged asymptomatic phase lasting several years, the disease usually manifests more visibly in adulthood. The GBD of CE is considerable, with an estimated annual burden exceeding 1 million DALYs. There are notable regional disparities, particularly in regions such as China, Central Asia, and Eastern Europe [1,6,32]. China ranks among the most affected nations, with high ASPR reported in regions such as Inner Mongolia, Sichuan, Tibet, Gansu, Qinghai, Ningxia, Yunnan, Shaanxi, and Xinjiang. The disease burden in China (398,000 DALYs) positions it at the forefront globally, necessitating targeted public health efforts [25]. Since 2005, China has gradually implemented a national CE control program developing a comprehensive strategy that includes infection source control, health education, management of intermediate host, and patient diagnosis and treatment [49–51].

In light of this context, our study conducts an in-depth analysis of the trends in the disease burden of CE among children and adolescents globally from 1990 to 2021. By employing a comprehensive set of methodologies, we revealed trends and health inequalities associated with CE across different regions and varying levels of the SDI worldwide, while also making predictions for future disease burdens.

Our examination of CE across regions with varying SDI levels highlighted several critical trends. High SDI regions exhibited relatively lower ASPR compared to low SDI regions, likely due to superior medical resources, improved living environments, and more extensive health education, also contributing to reduced disease incidence. Notably, the incidence of CE is higher among younger populations, gradually decreasing with age, this trend may stem from younger individuals engaging in activities that increase their exposure to infection sources.

ASMR further illustrate disparities associated with CE. Significant disparities exist between different SDI locations, with higher ASMR reported in low SDI areas, particularly among the elderly population aged 75 and older, likely due to challenges in accessing appropriate medical care. Indicators such as DALYs, YLDs, and YLLs tend to be higher among young populations, indicating that CE exerts a more severe impact on the younger generatio and may hinder their long-term health and productivity [52]. The elevated values for these indicators in children and adolescents may be related to their limited self-protection abilities, lack of health knowledge, and immature immune systems [1].

In 2021, a total of 101,105 cases of CE among children and adolescents were reported worldwide, representing a modest decrease of only 1.59% compared to 1990. This slight reduction underscores the persistent severity of the issue, positioning CE as a critical public health concern among adolescents. Additionally, considerable regional disparities in the ASPR were noted. In 2021, the global ASPR was 3.71 cases, whereas low SDI regions reported 6.48 cases, highlighting stark inequalities in disease burden. These disparities reflect the profound impact of socioeconomic development on health outcomes and highlight the pressing issue of global health inequities [16,31].

While low SDI regions continue to confront significant public health challenges, the notable decline in the burden of CE among children and adolescents deserves attention. This trend may correlate with strengthed public health interventions, widespread health education, and advancements in global medical technology. Since 2000, the ongoing decline in CE burden has become increasingly apparent, reflecting a growing commitment within the global health community to enhance the control of infectious and parasitic diseases, including CE [53,54].

Our findings reveal significant health inequalities between different socioeconomic levels, and groups with lower socioeconomic status bear a disproportionate disease burden. From 1990 to 2021, the slope index of ASPR for CE in children and adolescents globally changed from -2.597 to -1.087, indicating a more pronounced decline in prevalence among low SDI regions. This health inequality emphasizes the necessity for future public health policies and resource allocation to prioritize these vulnerable populations, aiming to reduce health disparities [25].

Moreover, we observed a negative correlation between the level of socioeconomic development and the disease burden of CE. As SDI improves, various health indicators associated with CE show a downward trend. We calculated the Pearson correlation coefficient and Spearman’s rank correlation coefficient between the ASR of CE and SDI for each year from 1990 to 2021 across 204 countries. For most indicators and years, the relationships exhibited different patterns. For instance, when the relationship appeared linear, the Pearson correlation was informative, while for non - linear patterns, the Spearman correlation was more robust. Taking the incidence indicator as an example, in 2008 and 2021, the Pearson correlation showed no significance (indicating a non - linear relationship), whereas the Spearman’s rank correlation still indicated a significant monotonic negative association (S4 Text). Overall, the Pearson correlation coefficient between the ASPR of CE and SDI had a pooled value of -0.253 (p < 0.0001), and the Spearman’s rank correlation coefficient was p = - 0.227(p < 0.0001), confirming a consistent tendency of negative association that is robust to potential non - linearity and heteroscedasticity in the data. The non - linear relationship was further visualized using a loess smoothing curve (S5 Text). This correlation analysis was performed by pooling global data across all study years (1990–2021) and also by analyzing each year individually. Given that the primary objective was to estimate the overall strength of the association and considering the consistent effect sizes across years (with Spearman results being more reliable in cases of non - linearity, such as the incidence indicator in 2008 and 2021), no correction for multiple testing was applied. The consistent effect sizes across both correlation measures and the highly significant p-values provide confidence in the reported relationship, indicating a significant decline in prevalence with increasing SDI. This finding emphasizes the importance of enhancing socioeconomic development to improve the health status of children and adolescents affected by CE globally [25].

Additionally, our study reveals the impact of population growth, aging, and epidemiological changes on the disease burden of CE. In low SDI regions, population growth emerges as the primary driver of the disease burden, while in middle SDI regions, the impact of epidemiological changes becomes more significant. This suggests that tailored prevention and control strategies should be adopted in regions with different levels of development.

Looking to the future, the disease burden is expected to continue their decline. However the wide prediction intervals for prevalence, incidence, and YLDs are wide indicate a high degree of uncertainty regarding future changes, influenced by factors, such as the distribution of healthcare resources and the efficacy of disease control measures control strategies [25,41]. It is anticipated that the disease burden of CE in children and adolescents worldwide will continue to decline, but specific values require further data for accurate determination.

In conclusion, our findings indicate an overall decline in the disease burden of CE among children and adolescents over the past three decades. This downward trend is anticipated to continue from 2021 to 2050, especially in terms of deaths, DALYs, and YLLs. These results suggest a promising reduction in the global burden of CE in children and adolescents. This decline may be related to the enhanced global control efforts targeting CE, following the release of the WHO’s roadmap for neglected tropical diseases [31].

This study acknowledges several limitations. The data sources from the GBD database encompass statistical information from national, regional, and international organizations, which introduces variability in data quality and collection methods across different regions. Such diversity in data quality may affect the consistency and comparability of the findings, potentially impacting the reliability of the analytical results. Additionally, GBD analyses often rely on a series of assumptions, especially when establishing causal relationships and constructing predictive models. These assumptions may not **apply** to all countries or regions, especially in contexts with significant differences in socioeconomic conditions, health policies, and cultural backgrounds, which may lead to biased results [36,37,40,41].

Our study provides valuable evidence for global health policymakers, enabling public health officials to develop and implement cost-effective intervention strategies to mitigate the disease burden of CE. We advocate for allocating additional health resources to children and adolescents to reduce their CE disease burden. Future research should investigate region-specific factors contributing to the CE disease burden and ways to enhance targeted public health interventions [28]. Continuous monitoring of the CE disease burden is essential to assess the effectiveness of current control measures and inform future public health policies with robust scientific evidence [30].

## Supporting information

S1 Table Posterior hyperparameters for the Bayesian Age-Period-Cohort (BAPC) model. (XLSX)

S2 Table Detailed CE burden indicators among children and adolescents by GBD regions (2021 and 1990–2021 Trends). (XLSX)

S3 Table Detailed CE burden indicators among children and adolescents in 204 countries (2021 and 1990–2021 Trends). (XLSX)

S1 Text Detailed formulas for SII and CI. (DOCX)

S2 Text R Code, data for the Bayesian Age-Period-Cohort (BAPC) model. (DOCX)

S3 Text Forecast validation metrics for the Bayesian Age-Period-Cohort (BAPC) model. (DOCX)

S4 Text Graph of temporal trends in correlations of related health indicators of cystic Echinococcosis. (DOCX)

S5 Text Non-linear association between age-standardized prevalence rate (ASPR) and Socio-demographic Index (SDI) visualized by LOESS smoothing. (DOCX)
