# Supplementary material for: Trends in the global burden of cystic echinococcosis among children and adolescents from 1990 to 2021: An analysis based on the Global Burden of Disease Study 2021
Source: PLoS Negl Trop Dis. 2025 Oct 30;19(10):e0013658. doi: 10.1371/journal.pntd.0013658 (PMC12574883; doi:10.1371/journal.pntd.0013658)
Supplement: S1 Text — (DOCX) [file pntd.0013658.s004.docx]

**S1 Text.** Detailed formulas for SII and CI.

**Slope Index of Inequality (SII)**

The SII quantifies the absolute difference in the outcome (age-standardized rate,$y$) between the most and least socioeconomically advantaged countries, estimated via weighted linear regression: SII=$\beta_{1}$​
where:

- $\beta_{1}$​ is the regression coefficient from the weighted linear model $y_{i}=\beta_{0}+\beta_{1}x_{i}+\epsilon_{i}$;
- $x_{i}$ is the fractional rank of country $ⅈ$ based on SDI (ranging from 0 for the lowest SDI to 1 for the highest SDI);
- $\epsilon_{i}$ is the error term;
- Weights are the annual population of country $i\left( \omega_{i} \right)$, with the regression weighted $\omega_{i}$ to account for population size.

**Concentration Index (CI)**

The CI quantifies relative socioeconomic inequality, calculated using the weighted covariance between the outcome $y_{i}$ and the fractional rank of SDI $x_{i}$:$CI=\frac{2}{\mu}\cdot\frac{\sum_{i=1}^{n} \omega_{i}\left( x_{i}-\bar{x}_{\omega} \right)\left( y_{i}-\bar{y}_{\omega} \right)}{\sum_{i=1}^{n} \omega_{i}}$

where:

- $\mu=\bar{y}_{\omega}=\frac{\sum_{i=1}^{n} \omega_{i}y_{i}}{\sum_{i=1}^{n} \omega_{i}}$ (population-weighted mean of the age-standardized rate);
- $\bar{x}_{\omega}=\frac{\sum_{i=1}^{n} \omega_{i}x_{i}}{\sum_{i=1}^{n} \omega_{i}}$ (population-weighted mean of the fractional SDI rank);
- $\omega_{i}$ = annual population of country $ⅈ$;

n = number of countries included in the analysis.
