# Supplementary material for: Trends in the global burden of cystic echinococcosis among children and adolescents from 1990 to 2021: An analysis based on the Global Burden of Disease Study 2021
Source: PLoS Negl Trop Dis. 2025 Oct 30;19(10):e0013658. doi: 10.1371/journal.pntd.0013658 (PMC12574883; doi:10.1371/journal.pntd.0013658)
Supplement: S2 Text — (DOCX) [file pntd.0013658.s005.docx]

**S2 Text.** R Code, Data for the Bayesian Age-Period-Cohort (BAPC) Model.
