# Supplementary material for: Trends in the global burden of cystic echinococcosis among children and adolescents from 1990 to 2021: An analysis based on the Global Burden of Disease Study 2021
Source: PLoS Negl Trop Dis. 2025 Oct 30;19(10):e0013658. doi: 10.1371/journal.pntd.0013658 (PMC12574883; doi:10.1371/journal.pntd.0013658)
Supplement: S5 Text — (DOCX) [file pntd.0013658.s008.docx]

**S5 Text.** Non-linear association between age-standardized prevalence rate (ASPR) and Socio-demographic Index (SDI) visualized by LOESS smoothing.





(A)Prevalence; (B)Incidence; (C)Deaths; (D)DALYs; (E)YLDs; (F) YLLs;
